# Co-morbidity of progressive supranuclear palsy and amyotrophic lateral sclerosis: a clinical-pathological case report

**DOI:** 10.1186/s12883-019-1402-7

**Published:** 2019-07-18

**Authors:** Koji Fujita, Tomoyasu Matsubara, Ryosuke Miyamoto, Hiroyuki Sumikura, Toshiaki Takeuchi, Keiko Maruyama Saladini, Toshitaka Kawarai, Hiroyuki Nodera, Fukashi Udaka, Kodai Kume, Hiroyuki Morino, Hideshi Kawakami, Masato Hasegawa, Ryuji Kaji, Shigeo Murayama, Yuishin Izumi

**Affiliations:** 1 Department of Neurology, Tokushima University Graduate School of Biomedical Sciences, 3-18-15 Kuramoto-cho, Tokushima, 770-8503 Japan; 2 Department of Neurology and Neuropathology (the Brain Bank for Aging Research), Tokyo Metropolitan Geriatric Hospital & Institute of Gerontology, 35-2 Sakae-cho, Itabashi-ku, Tokyo, 173-0015 Japan; 3 Pressent address: PVO Japan, Patient Safety Japan, Regulatory Office Japan, Novartis Pharma K.K., 1-23-1 Toranomon, Minayo-ku, Tokyo, 105-6333 Japan; 4 Department of Neurology, Sumitomo Hospital, 5-3-20 Nakanoshima, Kita-ku, Osaka, 530-0005 Japan; 5 Department of Epidemiology, Research Institute for Radiation Biology and Medicine, Horoshima University, 1-2-3 Kasumi, Minami-ku, Hiroshima, 734-8553 Japan; 6 Department of Dementia and Higher Brain Function, Tokyo Metropolitan Institute of Medical Science, 2-1-6 Kamikitazawa, Setagaya-ku, Tokyo, 156-8506 Japan

**Keywords:** Amyotrophic lateral sclerosis, Progressive supranuclear palsy, TAR DNA-binding protein 43 kDa (TDP-43), Tau, Copathology

## Abstract

**Background:**

The coexistence of distinct neurodegenerative diseases in single cases has recently attracted greater attention. The phenotypic co-occurrence of progressive supranuclear palsy (PSP) and amyotrophic lateral sclerosis (ALS) has been documented in several cases. That said, the clinicopathological comorbidity of these two diseases has not been demonstrated.

**Case presentation:**

A 77-year-old man presented with gait disturbance for 2 years, consistent with PSP with progressive gait freezing. At 79 years old, he developed muscle weakness compatible with ALS. The disease duration was 5 years after the onset of PSP and 5 months after the onset of ALS. Neuropathological findings demonstrated the coexistence of PSP and ALS. Immunohistochemical examination confirmed 4-repeat tauopathy, including globose-type neurofibrillary tangles, tufted astrocytes, and oligodendroglial coiled bodies as well as TAR DNA-binding protein 43 kDa pathology in association with upper and lower motor neuron degeneration. Immunoblotting showed hyperphosphorylated full-length 4-repeat tau bands (64 and 68 kDa) and C-terminal fragments (33 kDa), supporting the diagnosis of PSP and excluding other parkinsonian disorders, such as corticobasal degeneration. Genetic studies showed no abnormalities in genes currently known to be related to ALS or PSP.

**Conclusions:**

Our case demonstrates the clinicopathological comorbidity of PSP and ALS in a sporadic patient. The possibility of multiple proteinopathies should be considered when distinct symptoms develop during the disease course.

**Electronic supplementary material:**

The online version of this article (10.1186/s12883-019-1402-7) contains supplementary material, which is available to authorized users.

## Background

Progressive supranuclear palsy (PSP) was originally described as involving dystonic posturing of the neck and axial rigidity, vertical supranuclear gaze palsy, postural instability, gait disturbance with an ataxic quality, early falls, dysarthria, dysphagia, and a poor levodopa response [1]. PSP is neuropathologically characterized by extensive degeneration of the globus pallidus, subthalamic nucleus, substantia nigra, and pons as well as 4-repeat tau accumulation in both neurons and glia [2, 3]. Broad clinical phenotypes of PSP have been reported, including Richardson’s syndrome, progressive gait freezing, corticobasal syndrome, and primary lateral sclerosis (PLS) [4]. The PSP-PLS phenotype presents with upper motor neuron involvement and isolated tau pathology without TAR DNA-binding protein 43 kDa (TDP-43) pathology [5, 6]. In contrast, amyotrophic lateral sclerosis (ALS) is not regarded as a phenotype of PSP.

The co-occurrence of PSP and ALS has recently attracted greater attention. The phenotypic coexistence of PSP and ALS has been documented in several cases [7–9]. Cases of ALS plus PSP-like syndrome due only to TDP-43 proteinopathy have also been reported [10]. These reports, however, fell short of demonstrating neuropathology to begin with or dual neuropathologies. Some PSP cases have accompanying TDP-43 pathology in the limbic system and PSP-vulnerable regions, such as the subthalamic nucleus, substantia nigra, and pontine tegmentum, but not in the motor neuron system [11]. Thus, the neuropathological co-occurrence of ALS and PSP has not been demonstrated. Here, we report a unique case in which the coexistence of PSP and ALS was demonstrated both clinically and pathologically.

## Case presentation

**Fig. 1:**
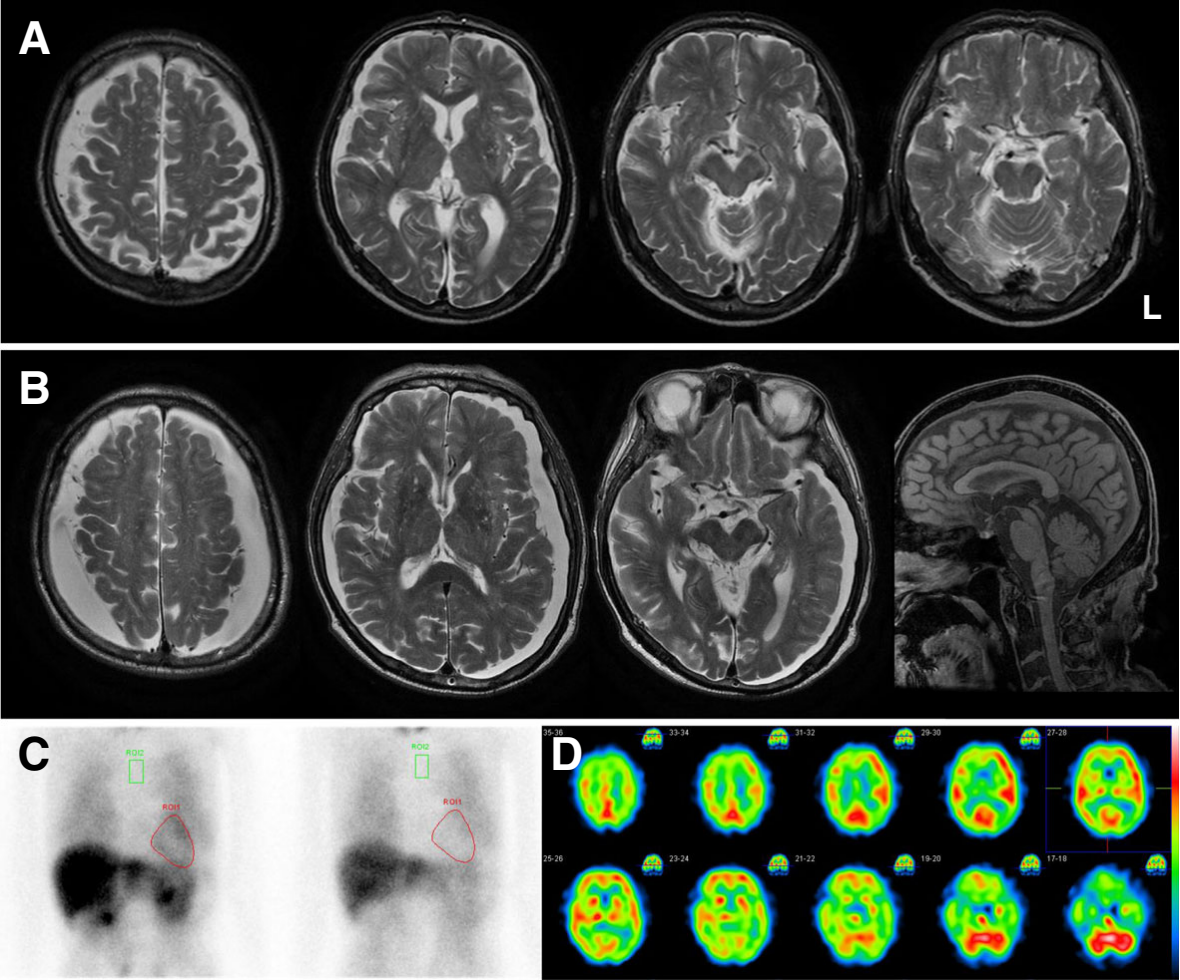
Imaging findings. **a** A brain MRI performed at 77 years old revealed mild frontal lobe atrophy and mild right-dominant subdural hygroma. **b** A follow-up MRI performed at 79 years old showed increased subdural hygroma and atrophy in the midbrain tegmentum. **c** Cardiac ^123^I-meta-iodobenzylguanidine scintigraphy showed decreased uptake in the delayed phase, suggesting mild sympathetic denervation. **d** Brain N-isopropyl-p-[^123^I]iodoamphetamine scintigraphy showed hypoperfusion in the bilateral frontal, parietal, and occipital lobes but not the basal ganglia, thalamus, and cerebellum

A 77-year-old man visited our hospital due to short-stepped gait and falls, which started two years before. He had a past history of herniated lumber disc, cataract, and benign prostatic hyperplasia. His family history was unremarkable. Neurological examination revealed bradykinesia, mildly reduced arm swing on the right when walking, and retropulsion. He did not present eye movement abnormalities, resting or postural tremors, apparent rigidity, or signs of autonomic impairment. Brain MRI revealed mild frontal lobe atrophy and mild right-dominant subdural hygroma (Fig. 1a). Levodopa/carbidopa hydrate was started but discontinued shortly due to side effects.

Nine months later, he was referred to us again due to forgetfulness and progressive gait disturbance. Neurological examination demonstrated scores of 26 and 16 in the Mini-Mental State Examination and Hasegawa Dementia Scale-Revised respectively, normal ocular and tongue movements and limb muscular strength, a positive snout reflex, a normal jaw jerk, increased tendon reflexes in the extremities, normal plantar response, short-stepped gait (especially on turning), retropulsion, and subsequent freezing of gait. Dysphagia, tremors, and rigidity were absent; muscular atrophy, fasciculation, and Hoffmann sign were not documented. A combination of amantadine, pramipexole, and levodopa/carbidopa hydrate, which was resumed, was partially effective for gait disturbance, but he still had falls and reported difficulty in writing. His body weight decreased from 64 kg to 42 kg in approximately one year. A follow-up MRI showed increased subdural hygroma and atrophy in the midbrain tegmentum (Fig. 1b).

Thirteen months after the second visit, when he was 79 years old, left- and proximal-dominant arm weakness was noted. A brain computed tomography revealed subdural hematoma predominantly on the left (Additional file 1). He was admitted to our hospital and underwent a burr-hole evacuation for the hematoma. Bilateral arm weakness and dysarthria progressed after the surgery. Neurological re-examination showed hypophonic dysarthria (which was not spastic), prominent muscle weakness in the shoulder girdle and upper limbs (manual muscle testing: deltoid 1/0, biceps 2/2, triceps 2/2, wrist flexor 5/5, wrist extensor 4−/2, and finger extensor 4/4−) but not the lower limbs (5/5), mild rigidity in the limbs (without spasticity), an increased jaw reflex, Hoffmann sign on the right, and equivocal plantar response bilaterally. Tendon reflexes were decreased in the left upper limb, mildly increased in the left lower limb, and otherwise normal. He had no restricted eye movement, dysphagia, tongue atrophy, apparent fasciculation, nuchal rigidity, or sensory disturbances. An electrophysiological study demonstrated active and chronic denervation with profound fasciculation potentials in the muscles of the brainstem, cervical, thoracic, and lumbar regions, fulfilling the definition of lower motor neuron dysfunction according to the updated Awaji criteria [12]. An ultrasonographic study revealed atrophy in the left cervical nerve roots and the left ulnar nerve and fasciculation in the upper and lower limbs. On respiratory function test (which was a poor study), %VC was 13.2 and FEV_1_% was 152.3. An arterial blood gas demonstrated pH 7.458, PaCO_2_ 46.7 mmHg, PaO_2_ 82.9 mmHg, HCO_3_ 32.3 mEq/L, and SaO_2_ 96.3%. Cardiac ^123^I-meta-iodobenzylguanidine scintigraphy showed decreased uptake in the delayed phase, suggesting mild sympathetic denervation. Brain N-isopropyl-p-[^123^I]iodoamphetamine scintigraphy showed hypoperfusion in the bilateral frontal, parietal, and occipital lobes but not the basal ganglia, thalamus, and cerebellum. He was diagnosed with clinically probable ALS according to the revised El Escorial and updated Awaji criteria, and riluzole was introduced. The score of ALS Functional Rating Scale-Revised was 27. CO_2_ retention worsened quickly, and dysphagia became apparent, requiring nasogastric tube nutrition. Due to the worsening of CO_2_ narcosis with aspiration pneumonia, he died at age 80 years old, five months after the development of muscle weakness.

We performed a genetic analysis that included whole-exome sequencing, and the results demonstrated that there was no pathological variation in genes currently known to be associated with PSP or ALS, including *MAPT* and *GRN*. No hexanucleotide repeat expansion was detected in *C9orf72*. The number of CAG repeats in the *ATXN2* gene was 25/25 (normal).

### Neuropathological findings

**Fig. 2:**
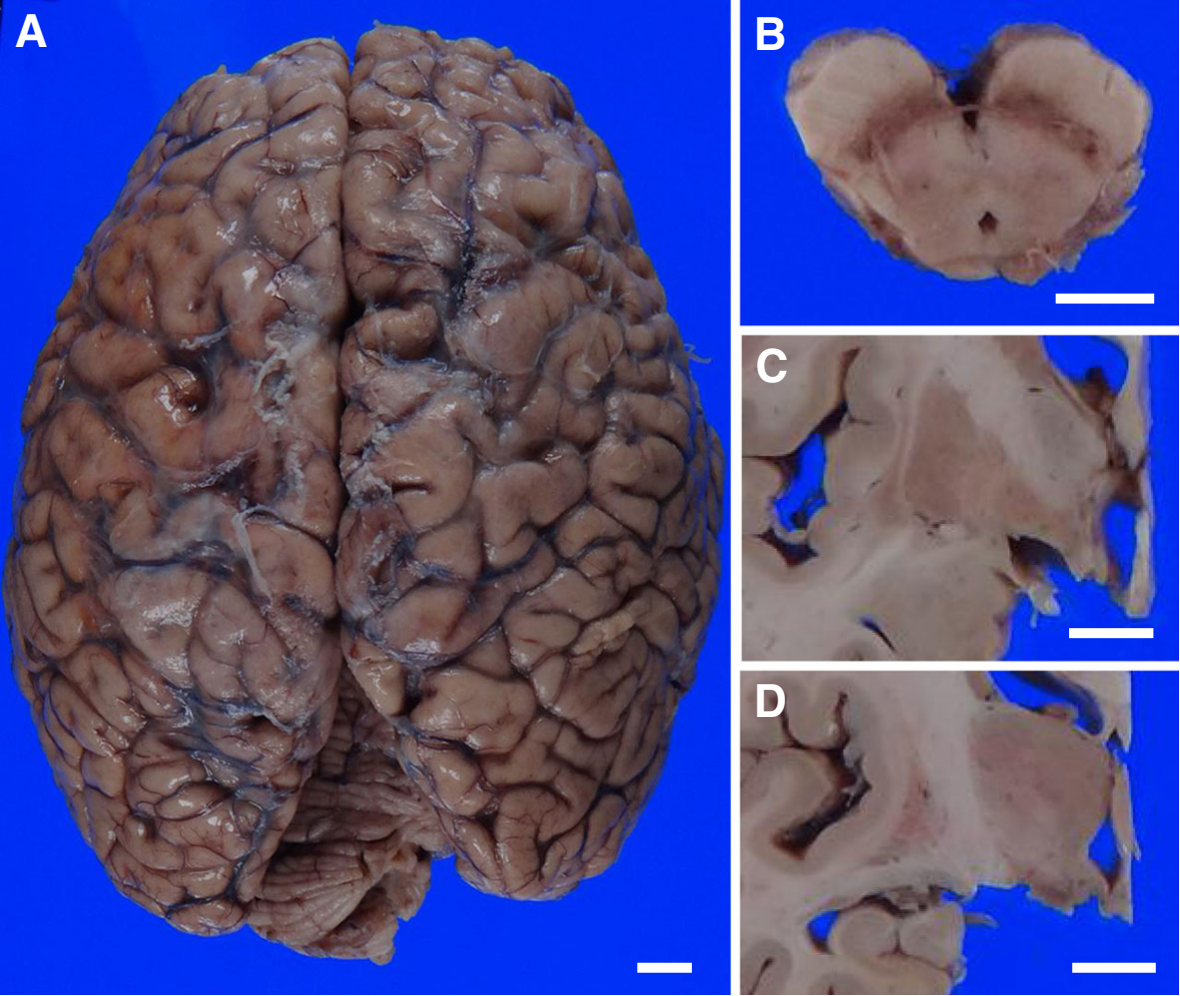
Macroscopic findings. The precentral gyrus showed mild atrophy (**a**). Depigmentation of the substantia nigra and atrophy of the tegmentum was observed in the midbrain (**b**). The internal segment of the globus pallidus (**c**) and the subthalamic nucleus (**d**) were atrophied. Scale bars: 1 cm (**a-d**)

The brain weight was 1,240 g. Macroscopic examination revealed selective atrophy of the anterior cervical nerve roots as well as mild atrophy of the precentral gyrus (Fig. 2a). In sections, depigmentation of the substantia nigra and atrophy of the tegmentum of the midbrain, internal globus pallidus and subthalamic nucleus were observed (Fig. 2b-d).

**Fig. 3:**
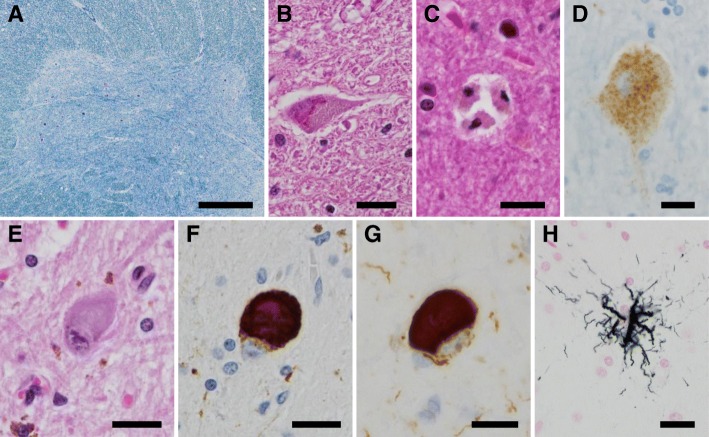
Microscopic findings. The cervical spinal cord showed severe neuronal loss of anterior horn cells (**a**) and Bunina bodies (**b**). Neuronophagia was observed in the precentral gyrus (**c**). Phosphorylated TDP-43 neuronal cytoplasmic inclusions were present in the precental gyrus (**d**). Globose-shaped neurofibrillary tangle (NFT) was detected in the substantia nigra by H-E staining (**e**) and immunostaining for phosphorylated tau (**f**) and 4-repeat tau (**g**). Tufted astrocytes were present in the putamen (**h**). Scale bars: 500 μm (**a**), 20 μm (**b-h**)

Microscopically, there was moderate to severe neuronal loss in the spinal anterior horn at all levels of the spinal cord (Fig. 3a) that relatively spares the Onufrowicz nuclei and Clarke’s column. Bunina bodies were abundant in anterior horn cells (Fig. 3b). Macrophages had aggregated in the corticospinal tracts, while myelin pallor was not evident. Mild to moderate neuronal loss and gliosis were observed in the hypoglossal nuclei. In the amygdala and area CA1 of the hippocampus, moderate to severe neuronal loss and gliosis were noted. Betz cells were mildly reduced, and some neuronophagia was observed in the precentral gyrus (Fig. 3c). On immunohistochemistry, we found that phosphorylated TDP-43-immunopositive neuronal cytoplasmic inclusions (NCIs) and glial cytoplasmic inclusions (GCIs) were predominantly located in the spinal anterior horn cells, brainstem motor nuclei (including the hypoglossal, facial, and trigeminal nuclei), the precentral gyrus, and the limbic systems (including the amygdala, hippocampus, subiculum, and entorhinal cortex) (Fig. 3d).

Moreover, the substantia nigra showed marked neuronal loss with astrogliosis, melanophagia, and globose-shaped neurofibrillary tangles (NFTs) (Fig. 3e). NFTs had accumulated in the oculomotor nuclei, while neuronal loss was not obvious. Mild neuronal loss and grumose degeneration were observed in the dentate nuclei of the cerebellum. Neuronal loss and gliosis with accumulation of NFTs were observed in the subthalamic nucleus and globus pallidus. In addition, adequate amounts of NFTs and coiled bodies, immunopositive for phosphorylated tau and 4-repeat tau, were observed in PSP-vulnerable areas, including the inferior olivary nucleus, substantia nigra, subthalamic nucleus, and globus pallidus (Fig. 3f, g). These structures showed less immunoreactivity for 3-repeat tau. Tufted astrocytes were abundant in the substantia nigra, red nucleus, midbrain tectum, subthalamic nucleus, putamen, caudate nuclei, and precentral gyrus (Fig. 3h). NFT, coiled bodies, and tufted astrocytes were also observed following Gallyas-Braak staining.

The phosphorylated TDP-43-positive structures and 4-repeat tau-positive structures did not colocalize, as investigated by double immunohistochemistry in the substantia nigra or precentral gyrus. Together, these findings demonstrate the neuropathological coexistence of ALS and PSP. Only a small number of NFTs that were immunopositive for both RD4 and RD3 were detected in restricted regions, such as the transentorhinal cortex. This pathological feature was consistent with Braak NFT stage Ι [13] and Braak AT8 stage Ι [14]. No deposition of amyloid, argyrophilic grain, or α-synuclein was detected.

**Fig. 4:**
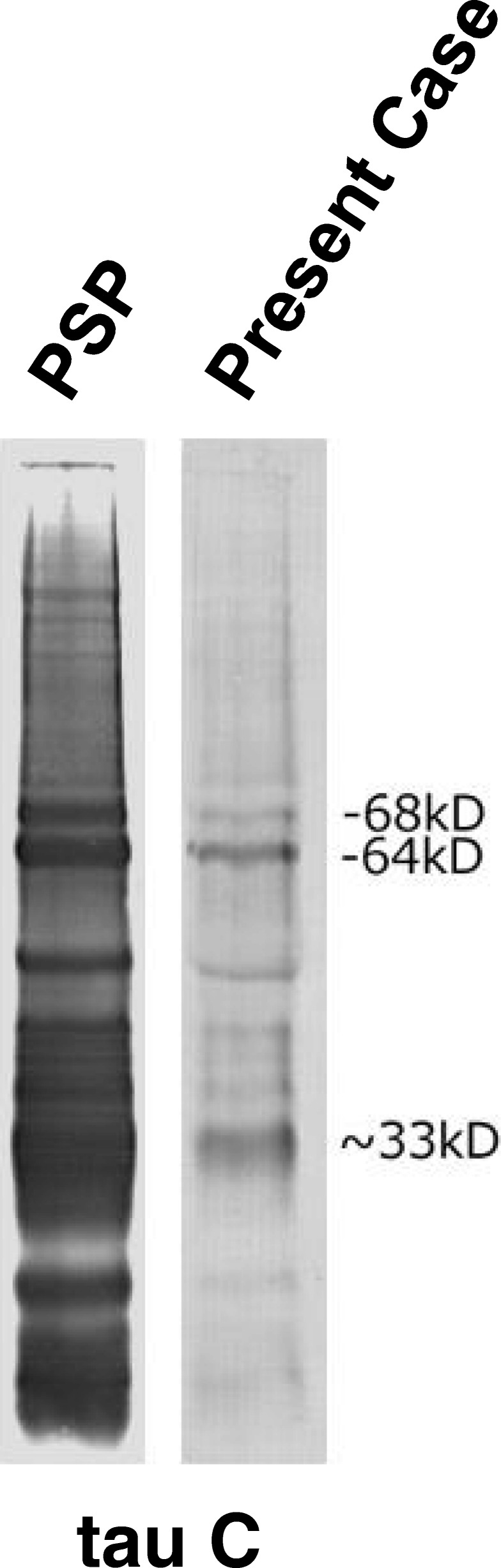
Immunoblot analysis of sarkosyl-insoluble tau. Full-length hyperphosphorylated tau bands indicated by 64, 68 kD and ~ 33 kDa fragments were detected, consistent with a progressive supranuclear palsy tau banding pattern

Immunoblotting showed hyperphosphorylated full-length 4-repeat tau bands (64 and 68 kDa) and C-terminal fragments (33 kDa) in the frontal cortex (Fig. 4). This banding pattern was consistent with that previously reported in PSP [15, 16].

The methods used in the above analyses are provided in Additional file 2.

## Discussion and conclusions

Here, we demonstrate a distinct case in which PSP and ALS coexisted clinically and pathologically. Clinically, our case corresponds to possible PSP with progressive gait freezing and probable ALS [4, 12, 17]; as in our case, variant PSP syndromes other than Richardson’s often lack vertical gaze palsy [4]. Pathological investigation demonstrated neuronal loss and degeneration in the regions typically affected by PSP and ALS. We also show that 4 repeat-tau accumulation was present in the PSP-associated regions, while TDP-43 accumulation was present in the ALS-related regions [3, 18], supporting the dual pathologies of PSP and ALS. Moreover, the biochemical findings of tau also confirmed the diagnosis of PSP and excluded corticobasal degeneration.

Given that both PSP (with Richardson’s syndrome; 3.1–13.8 per 100,000 persons [19–21]) and ALS (5.0–10.3 per 100,000 persons [22–24]) are rare, the accidental comorbidity of PSP and ALS is highly unlikely. Rather, our case may accord with the high frequency of multiple pathologies in single cases, as revealed by recent advances in immunohistochemical methods [25, 26]. PSP sometimes accompanies other neurodegenerative pathologies, although they do not necessarily correspond to clinical symptoms. For instance, a PSP brain is apparently accompanied by TDP-43 pathology more often than a normal aging brain [11, 26, 27]. Furthermore, there have been reports of cases with both PSP and other neurodegenerative pathologies, such as Alzheimer’s disease [28, 29], Lewy body disease [30], and ballooned neurons due to argyrophilic grain disease [31]. Cases of PSP with multiple system atrophy [32, 33] and Pick bodies [34] have also been reported.

Conversely, tau pathology can be observed in ALS or frontotemporal lobar degeneration with TDP-43 pathology (FTLD-TDP). In one study, tau pathology of Braak NFT stage III or higher, which is apparently related to Alzheimer’s pathology, was observed in over 15% of ALS and FTLD-TDP cases [26]. In addition, co-occurrence of argyrophilic grain disease, in which phosphorylated 4-repeat tau is the pathological protein, was documented in approximately 40% of ALS cases [35]. These findings indicate that the coexistence of 3- or 4-repeat tauopathies in ALS and FTLD-TDP is not uncommon. Of further note, the comorbidity of PSP-tau and FTLD-TDP has been reported in two cases. However, these cases lacked ALS-associated symptoms, such as muscle weakness and atrophy, or pathological changes in the motor neuron system [36]. Accordingly, our case represents a unique example of the clinical and pathological comorbidity of PSP and ALS.

That being said, as PSP per se can involve mild neuronal loss and NFTs in the spinal anterior horn, which possibly present as clinical symptoms [37], one may argue that the progressive weakness observed in our case could also be attributed to PSP pathology alone. In our case, however, neuronal loss and accumulation of TDP-43 inclusions were obvious in the spinal anterior horn, while NFTs and phosphorylated tau accumulation were scarce there. The distribution patterns of TDP-43 vs. tau pathologies were also found in the hypoglossal and facial nuclei, consistent with ALS pathology. Accordingly, the progressive weakness was likely due to the concurrent ALS pathology rather than phenotypic variation of PSP.

We note that there was little regional overlap between the ALS and PSP pathologies. Even in some regions in which both tau and TDP-43 pathologies were abundant (e.g., the precentral gyrus and substantia nigra), the severity of neuronal loss was similar to that observed in typical cases of PSP or ALS. Furthermore, we found no colocalization of 4-repeat tau and phosphorylated TDP-43 on double immunohistochemistry. These findings do not support the notion that mutual exacerbation of the dual pathologies occurred in our case. That said, the co-occurrence per se may have contributed to the short duration of ALS observed in our case (5 months).

We are aware that the combination of ALS and parkinsonism is a feature of the ALS-parkinsonism-dementia complex in the Kii peninsula of Japan (Kii ALS/PDC), in which the disease presents with NFTs of Alzheimer’s type without amyloid deposition, TDP-43 pathology, and α-synuclein pathology to varying degrees. However, the Kii ALS/PDC lacks tufted astrocytes [38], which we observed in our case. In addition, the Kii ALS/PDC showed hyperphosphorylated tau triplet bands at 60, 64, and 68 kDa on immunoblotting [39, 40]. Our case showed no 60 kDa band, in contrast with the Kii ALS/PDC. Taken together, we suggest that the neuropathological findings in our case are distinct from those of the Kii ALS/PDC.

We note that genetic abnormalities can be associated with PSP, ALS, and related disorders. For instance, *C9orf72* repeat expansions, which account for a large proportion of ALS and FTLD cases, are a rare genetic cause of parkinsonism, such as Parkinson’s disease, corticobasal syndrome, and PSP [41]. Mutations in *MAPT* and *GRN* have also been detected in PSP, CBS, and FTLD [42], and *TBK1* mutations can manifest as ALS, FTLD, or PSP [43]. Furthermore, genetic abnormalities in *C9orf72* versus *GRN* are associated with the degree of NFT tau pathology in FTLD, suggesting a role for *C9orf72* mutations in multiple proteinopathies [44]. However, in our case, the genetic studies, which included whole-exome sequencing, excluded genetic abnormalities known to be associated with these disorders.

The findings reported in our case show that distinct phenotypes, PSP and ALS, and their corresponding pathologies, including tauopathy and TDP-43 proteinopathy, can occur in a single case. Clinical symptoms distinct from those of the original syndromes, e.g., muscle atrophy and weakness in the course of atypical parkinsonism, should alert clinicians to the possibility of overlapping pathologies. It is crucial to recognize that overlapping pathologies can occur in single cases as distinct therapies become available or are being developed for distinct diseases, e.g., riluzole and edaravone for ALS and anti-tau immunotherapies for PSP. Furthermore, future clinical trials may have to consider the possibility of multiple pathologies to appropriately assess the efficacy of drug candidates.

## Additional files

Additional file 1: Brain computed tomography. (PPTX 3124 kb)

Additional file 2: Methods. (DOCX 22 kb)

## Data Availability

The data that support the findings presented in this study are available from the corresponding author upon reasonable request.

## Abbreviations

AC-PC: Anterior commissure-posterior commissure
ALS: Amyotrophic lateral sclerosis
ALS-PDC: ALS-parkinsonism-dementia complex
FTLD-TDP: Frontotemporal lobar degeneration with TDP-43 pathology
GCI: Glial cytoplasmic inclusion
HE: Hematoxylin-eosin
KB: Klüver-Barrera
NCI: Neuronal cytoplasmic inclusion
NFT: Neurofibrillary tangle
PLS: Primary lateral sclerosis
PSP: Progressive supranuclear palsy
TDP-43: TAR DNA-binding protein 43 kDa
