# Supplementary figures and images for: Co-morbidity of progressive supranuclear palsy and amyotrophic lateral sclerosis: a clinical-pathological case report
Source: BMC Neurol. 2019 Jul 18;19:168. doi: 10.1186/s12883-019-1402-7 (PMC6637486; doi:10.1186/s12883-019-1402-7)

## Slide 1
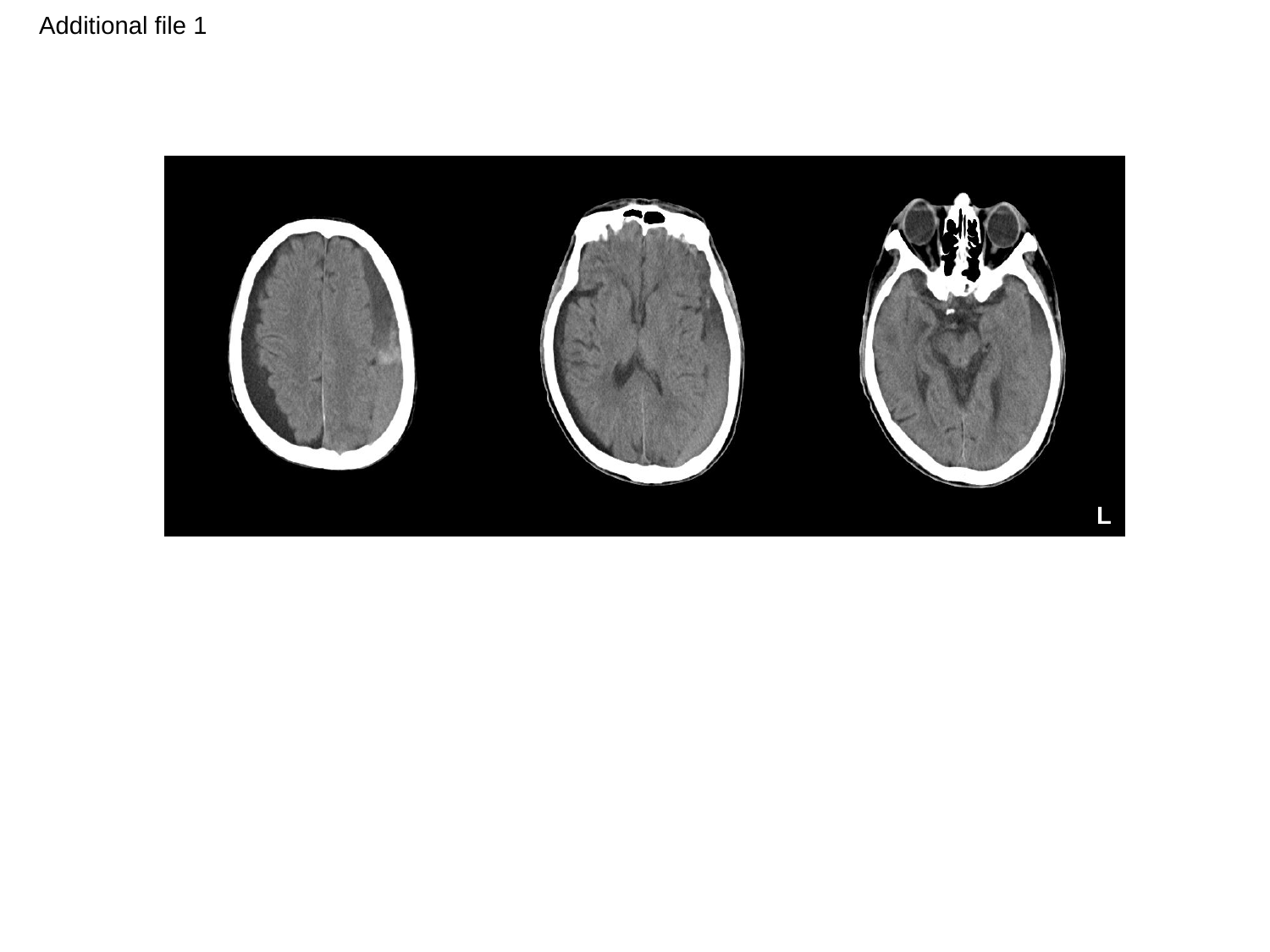

Additional file 1
L

Supplement: Supplementary file 1 — Brain computed tomography. (PPTX 3124 kb) [file 12883_2019_1402_MOESM1_ESM.pptx]
