# Supplementary material for: Co-morbidity of progressive supranuclear palsy and amyotrophic lateral sclerosis: a clinical-pathological case report
Source: BMC Neurol. 2019 Jul 18;19:168. doi: 10.1186/s12883-019-1402-7 (PMC6637486; doi:10.1186/s12883-019-1402-7)
Supplement: Supplementary file 2 — Methods. (DOCX 22 kb) [file 12883_2019_1402_MOESM2_ESM.docx]

**Additional file 2**

**Neuropathological examination**

The brain and spinal cord were fixed in 20% buffered formalin and embedded in paraffin. The right cerebral hemisphere was dissected in the coronal plane. The left hemisphere above the anterior commissure-posterior commissure (AC-PC) line was dissected in the sagittal plane for sufficient observation of the precentral gyrus, as previously described [1]. The left hemisphere below the AC-PC line was dissected in the axial plane. The brainstem and right cerebellum were dissected in the axial plane, and the left cerebellum was dissected in the sagittal plane. Six-micrometer-thick sections were stained with HE, KB, and Gallyas-Braak staining. Furthermore, we performed immunostaining with a Ventana BenchMark GX autostainer (Ventana Medical Systems, Tucson, AZ, USA), an I-View Universal DAB Detection Kit (Roche, Basel, Switzerland), and primary antibodies against phosphorylated tau (AT8; Innogenetics, Gent, Belgium), tau 4-repeat isoform RD4 (1E1/A6; Temecula, CA, USA), tau 3-repeat isoform RD3 (8E6/C11; Upstate, Lake Placid, NY, USA), phosphorylated TDP-43 (pSer409/410; a gift from M. Hasegawa, Japan), human amyloid β (12B2; IBL, Maebashi, Japan), and phosphorylated α-synuclein (pSyn#64; a gift from T. Iwatsubo, Japan).

**Immunoblot analysis**

Frozen tissue was obtained from the right frontal lobe and prepared for western blotting with an antibody to the human tau C-terminal region 429-441 (Tau C; rabbit polyclonal, dilution 1:1000, Cosmo Bio, Tokyo, Japan) according to previously described methods [2].

**Genetic analysis**

This investigation was approved by the institutional ethics committees of Tokushima University and Hiroshima University. Written informed consent was obtained from the patient. Genomic DNA was extracted from patient-derived peripheral lymphocytes. We chose 10 ALS genes to investigate: *FUS*, *SQSTM1*, *SOD1*, *VCP*, *SIGMAR1*, *TARDBP*, *OPTN*, *ANG*, *PFN1*, and *UBQLN2*. All coding exons were simultaneously PCR-amplified and sequenced. The large repeat expansion in the *C9orf72* gene was assessed using repeat-primed PCR [3]. Subsequently, whole-exome sequencing data analysis was performed.

**References**

1. Matsubara T, Oda M, Takahashi T, Watanabe C, Tachiyama Y, Morino H, Kawakami H, Kaji R, Maruyama H, Murayama S *et al*: **Amyotrophic lateral sclerosis of long clinical course clinically presenting with progressive muscular atrophy**. *Neuropathology* 2019, **39**(1):47-53.

2. Taniguchi-Watanabe S, Arai T, Kametani F, Nonaka T, Masuda-Suzukake M, Tarutani A, Murayama S, Saito Y, Arima K, Yoshida M *et al*: **Biochemical classification of tauopathies by immunoblot, protein sequence and mass spectrometric analyses of sarkosyl-insoluble and trypsin-resistant tau**. *Acta Neuropathol* 2016, **131**(2):267-280.

3. Cleary EM, Pal S, Azam T, Moore DJ, Swingler R, Gorrie G, Stephenson L, Colville S, Chandran S, Porteous M *et al*: **Improved PCR based methods for detecting C9orf72 hexanucleotide repeat expansions**. *Mol Cell Probes* 2016, **30**(4):218-224.
